# First report in italy of the exotic mosquito species *Aedes (Finlaya) koreicus*, a potential vector of arboviruses and filariae

**DOI:** 10.1186/1756-3305-4-188

**Published:** 2011-09-28

**Authors:** Gioia Capelli, Andrea Drago, Simone Martini, Fabrizio Montarsi, Mauro Soppelsa, Nicola Delai, Silvia Ravagnan, Luca Mazzon, Francis Schaffner, Alexander Mathis, Marco Di Luca, Roberto Romi, Francesca Russo

**Affiliations:** 1 Istituto Zooprofilattico Sperimentale delle Venezie, Laboratory of Parasitology, Viale dell'Università 10, Legnaro, 35020, Italy; 2 Entostudio, Via Buffa 9, Brugine, 35020, Italy; 3 Local Health Unit (ULSS 2), Department of Prevention, Via Borgo Ruga 30, Feltre, 32032, Italy; 4 University of Padua, Department of Environmental Agronomy and Crop Science, Viale dell'Università 16, Legnaro, 35020, Italy; 5 University of Zurich, Institute of Parasitology, Winterthurerstr. 266a, Zurich, 8057, Switzerland; 6 Department of Infectious, Parasitic and Immuno-mediated Diseases, Vector-Borne Diseases and International Health Unit, Istituto Superiore di Sanità, Viale Regina Elena 299, Rome, 00161, Italy; 7 Veneto Region, Direzione Prevenzione, Servizio Promozione e Sviluppo Igiene e Sanita' Pubblica, Dorsoduro 3493, Venice, 30123, Italy

**Keywords:** *Aedes koreicus*, Italy, exotic mosquito, invasive species, entomological monitoring

## Abstract

**Background:**

In the Veneto region (north-eastern Italy) an entomological surveillance system has been implemented since the introduction of the Asian tiger mosquito (*Aedes albopictus*) in 1991. During the routine monitoring activity in a tiger mosquito-free area, an unexpected mosquito was noticed, which clearly did not belong to the recorded Italian fauna.

**Findings:**

At the end of May 2011, twelve larvae and pupae were collected in a small village in Belluno province (Veneto region) from a single manhole. Ten adults reared in the laboratory were morphologically and genetically identified as *Aedes (Finlaya) koreicus *(Edwards, 1917), a species native to Southeast Asia. The subsequent investigations carried out in the following months in the same village provided evidence that this species had become established locally. Entomological and epidemiological investigations are currently ongoing in the surrounding area, to verify the eventual extension of the species outside the village and to trace back the route of entry into Italy.

**Conclusions:**

This is the first report in Italy of the introduction of the exotic mosquito *Ae. koreicus*. This species has been shown experimentally to be competent in the transmission of the Japanese encephalitis virus and of the dog heartworm *Dirofilaria immitis *and is considered a potential vector of other arboviruses. Thus, the establishment of this species may increase the current risk or pose new potential threats, for human and animal health. This finding considerably complicates the entomological monitoring of the Asian tiger mosquito *Ae. albopictus *in Italy and stresses the importance of implementing the entomological surveillance for the early detection of and the rapid response against invasive mosquito species.

## Background

After the introduction and establishment of the Asian tiger mosquito (*Aedes albopictus) *in north-eastern Italy in 1991 [1], an entomological surveillance, promoted by the Public Health Service of the Veneto region, was started. The entomological monitoring primarily relies on the use of ovitraps in the areas where the tiger mosquito is endemic, while in non-colonized areas, collection of larvae/pupae and adult trapping are carried out. In addition, information and education is provided targeting municipalities and Local Health Units as well as residents [2]. During the routine surveillance activity in a tiger mosquito-free area, an unexpected mosquito was noticed which clearly did not belong to the recorded Italian fauna.

### Mosquito findings and identification

**Figure 1:**
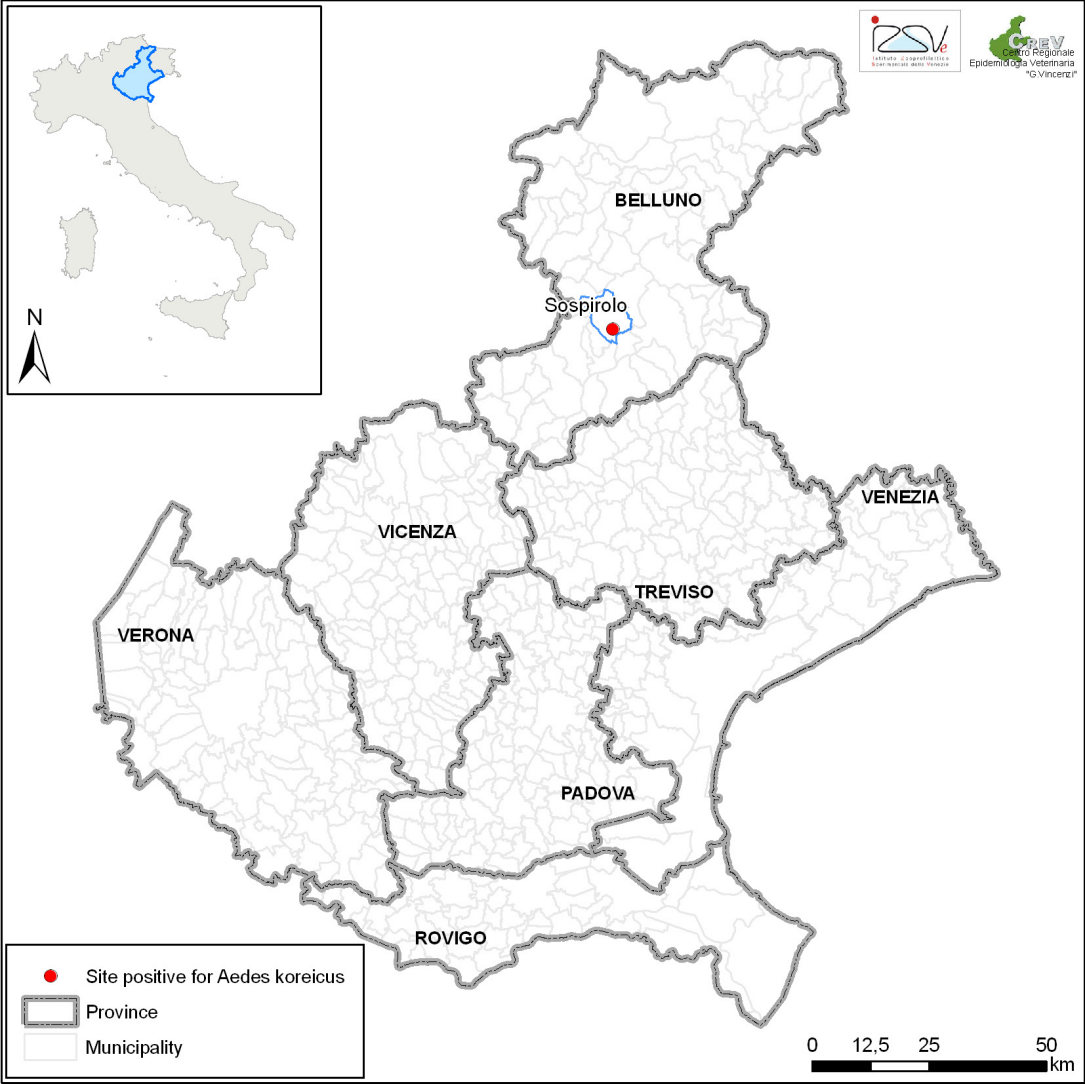
**Map showing the locality of the first *Aedes koreicus *finding in north-eastern Italy**.

At the end of May 2011, twelve larvae and pupae were collected in a small village in Belluno province, located at 447 m.a.s.l. (Lat 46° 8'30.27"N; Long 12° 4'19.33"E) (Figure 1). The larvae and pupae were collected from a single manhole.

The immature stages collected were reared in the laboratory and the ten adults obtained were morphologically identified, using taxonomic keys [3-6], as *Aedes (Finlaya) koreicus *(Edwards, 1917). Further investigations were carried out based on previously described morphological variations [5, Versteirt *et al*., pers. comm ], which had been observed on specimens from Belgium. In particular, the presence of a basal pale band on hind tarsomere V, led us to attribute the Italian specimens to the morphological form reported from Jeju-do, an island located in the Korea Strait, south of the peninsula. Due to this unexpected finding and considering that *Ae. koreicus *is closely related to *Ae. japonicus*, a biomolecular confirmation was considered appropriate. Three PCR assays were performed in different laboratories. DNA was amplified at three mitochondrial loci, two at the nicotinamide adenine dinucleotide dehydrogenase subunit 4 (ND4) gene and one at the cytochrome oxidase subunit II (COII) gene. The PCR protocols followed the methods of Cameron *et al*. [7], Uribe Soto *et al*. [8] and Simon *et al*. [9], respectively.

Amplicons were then sequenced and compared with GenBank entries. Thus, identities ranging from 99.6% to 99.7% with sequences attributed to *Ae. koreicus *(GenBank accession numbers: GU229897.1, GU229925, GU229926 and GU229927) were observed, confirming the morphological identification. At the mitochondrial loci, the intra-specific range of nucleotide differences is reported to be 0.2% and the inter-specific percent difference (among *Ae. koreicus *and the subspecies in the *Ae. japonicus *complex) ranges from 4.4% to 9.2% [7].

After the biomolecular confirmation, the village was checked again for the presence of the mosquito on July 17^th^. All the possible breeding sites, mainly public and private small water containers, were examined. Four additional sites were found positive for larvae of *Ae. koreicus*, namely two manholes, one bucket in a private garden and two flower pots at the cemetery, all included in a range of one km^2^. In particular the private garden was heavily infested, with thousands of larvae in the bucket of clean water. Approximately 300 larvae were collected, partly mounted on a slide and partly let to moult into adults. All the larvae and 40 adults were confirmed as *Ae. koreicus*. No other mosquito species were found to share the same breeding sites.

These new findings indicated that the species was already established in the village, although with a very limited spatial distribution. The territory of the municipality extends from 305 to 2.149 m.a.s.l. and is characterised by a temperate climate, with cold and often snowy winters and mild warm summers.

## Discussion and conclusions

*Ae. koreicus *is an Asian species native to Korea, Japan, China and eastern Russia. Not much information is available on the biology and activity of this species. It is reported to feed on humans and domestic animals, and it seems to be well adapted to the urban environment [5]. Larvae develop in all types of artificial containers close to houses, and even in pools on rocks in the hills or in tree holes. The adults seem to bite humans both during the day and at night. Like other species of the genus *Aedes*, *Ae. koreicus *overwinters in the egg stage, hatching in the spring when the snow melts [3,4]. As compared to the Asian tiger mosquito, *Ae. koreicus *seems to be more tolerant to a cold climate, making this species capable of surviving and becoming established in the hilly and pre-alpine areas of Italy.

Indeed, this species has previously been identified in Belgium in 2008, where it successfully established [10]. These are also the first findings of *Ae. koreicus *outside its native range, demonstrating its ability to establish and colonize new areas in a temperate climate, as previously suggested by Cameron *et al*. [7] who commented: "It is perhaps surprising, or just a matter of chance, that the most recent introduction to the United States was *Ae. j. japonicus *and not *Ae. koreicus *" and "there is the distinct possibility that *Ae. koreicus *will also begin spreading across the world".

It is currently difficult to speculate about the time of arrival of the mosquito in this part of Italy. Certainly, the species was not present or went unnoticed in the 2009-2010 summer surveillance at this location, where only a few adult specimens of *Ae. geniculatus *were identified.

Entomological and epidemiological surveys at the village and the surrounding area are ongoing in order to understand whether the species is already established outside the village and to attempt to trace back the possible route of entry. Morphological analysis led to the assumption that Jeju-do was the possible origin of the introduced population, and then international trade with this Korean province might be the cause for this introduction. Alternatively, connection with export companies from the area where *Ae. koreicus *is established in Belgium could be considered as a possible route of entry. Investigations on invasive mosquito species introduced into the USA and Europe (i.e. *Ae. albopictus *and *Ae. japonicus*) suggest that used tires and plant cuttings were the major vehicles [11]. Discarded tires are a common breeding habitat for many mosquito species, as shown for the USA by Yee [12], who documented 32 such mosquito species in his review of the literature over the last 50 years. Recently, Scholte et al. [13], during routine mosquito surveillance inspections at companies that import used tires in The Netherlands, reported the detection of three invasive species within a few months: the yellow fever mosquito (*Ae. aegypti*), the Asian tiger mosquito (*Ae. albopictus*), and the American rock-pool mosquito (*Ae. atropalpus*), demonstrating how frequently an exotic mosquito may be introduced.

In Italy in the past 20 years, invasive mosquito species were detected three times, namely *Ae. albopictus *in 1990 and 1991 [14,1], now endemic all over the country, *Ae. atropalpus *in 1996 [15], detected in Treviso province and promptly eradicated, and currently *Ae. koreicus*. The first two introductions were due to infested used tires from USA [15,16]. Interestingly, all these introductions occurred in different provinces of the same area of north-eastern Italy (Veneto Region). It is not clear whether this is due to the intense local active surveillance of the tiger mosquito or to the trade of goods, possibly infested. Indeed, north-eastern Italy is regarded as one of the most developed industrial and commercial area of the country.

**Figure 2:**
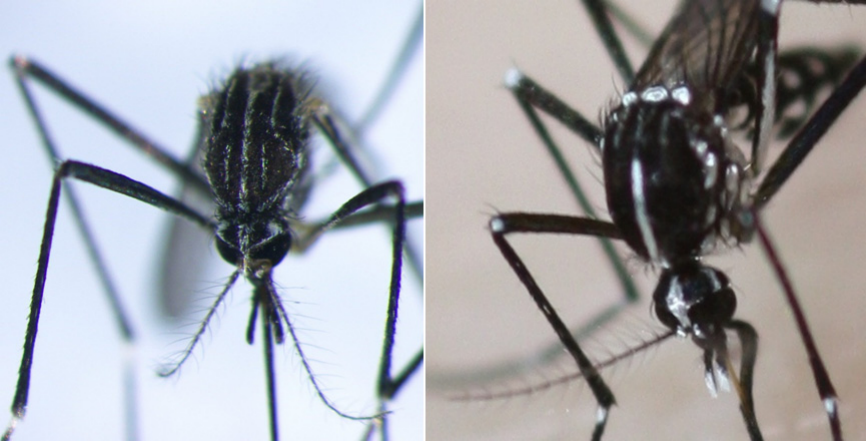
**Particular of the mesonotum of *Aedes koreicus *(left) compared to *Ae. albopictus *(right)**.

The possible establishment of *Ae. koreicus *in regions where the tiger mosquito is endemic would complicate the current entomological surveillance system of *Ae. albopictus*, which is mainly based on the use of inexpensive ovitraps. The detection of the typical black *Aedes *eggs in ovitraps, easy recognizable by non-expert personnel, was sufficient to determine the infestation status of a location with regard to the tiger mosquito. Unfortunately, tiger mosquito eggs are indistinguishable by simple observation under a binocular microscope from the eggs of other *Aedes *spp., including those of *Ae. koreicus*. Hence, the species identification at a routine basis in areas where *Ae. albopictus *and *Ae. koreicus *may share the same breeding sites, will require to rear the eggs in the laboratory or to include into the surveillance system the more laborious systematic collection of larvae and adults, which require an equipped laboratory and well trained personnel for identification (Figure 2).

*Ae. koreicus *is considered a potential vector of arboviruses [7], albeit the published evidence for its vector status is very scant. Many authors [3-5] argue that the species has been probably misidentified as *Ae. japonicus *in the past, thus confounding the vector competence of these two mosquitoes. *Ae. koreicus *has been, however, reported to be involved in the transmission of the Japanese encephalitis virus (JEV) [17], exotic to Europe, and of the dog heartworm *Dirofilaria immitis *[4].

Dog heartworm is currently endemic in the lowlands of north-eastern Italy [18], but it's seldom reported in hilly areas, like the one where *Ae. koreicus *was detected. Thus, its establishment may increase the current risk or pose new potential threats, for human and animal health. Certainly, vector competence studies are strongly required to better define the role of this mosquito species in the transmission of JEV and *D. immitis *and of other arboviruses, such as West Nile and USUTU viruses, both endemic in Veneto region [19].

This finding, once again, stresses the importance to implement the entomological surveillance for early detection of invasive species, which is imperative to prevent new establishments and to have a chance to promptly eradicate them.

## Competing interests

The authors declare that they have no competing interests.

## Authors' contributions

GC wrote the paper; FM, AD, SM, ND and MS where involved in the mosquito collection and laboratory rearing; FS, RR, FM and MDL morphologically identified the specimens; SR, LM and AM genetically identified the specimens; FR provided part of the funding; all the Authors, and especially RR, FS and AM, revised the MS and contributed with comments and suggestions. All authors read and approved the final version of the manuscript.
